# Heterogeneous Conservation of *Dlx* Paralog Co-Expression in Jawed Vertebrates

**DOI:** 10.1371/journal.pone.0068182

**Published:** 2013-06-28

**Authors:** Mélanie Debiais-Thibaud, Cushla J. Metcalfe, Jacob Pollack, Isabelle Germon, Marc Ekker, Michael Depew, Patrick Laurenti, Véronique Borday-Birraux, Didier Casane

**Affiliations:** 1 Institut des Sciences de l’Evolution, Université de Montpellier II, UMR5554, Montpellier, France; 2 Laboratoire Evolution Génome et Spéciation UPR9034 CNRS, Gif-sur-Yvette, France; 3 Center for Advanced Research in Environmental Genomics, University of Ottawa, Ottawa, Canada; 4 Department of Orthopaedic Surgery, University of California San Francisco, San Francisco, California, United States of America; 5 Université Paris Diderot, Paris, France; University of Lausanne, Switzerland

## Abstract

**Background:**

The *Dlx* gene family encodes transcription factors involved in the development of a wide variety of morphological innovations that first evolved at the origins of vertebrates or of the jawed vertebrates. This gene family expanded with the two rounds of genome duplications that occurred before jawed vertebrates diversified. It includes at least three bigene pairs sharing conserved regulatory sequences in tetrapods and teleost fish, but has been only partially characterized in chondrichthyans, the third major group of jawed vertebrates. Here we take advantage of developmental and molecular tools applied to the shark *Scyliorhinus canicula* to fill in the gap and provide an overview of the evolution of the *Dlx* family in the jawed vertebrates. These results are analyzed in the theoretical framework of the DDC (Duplication-Degeneration-Complementation) model.

**Results:**

The genomic organisation of the catshark *Dlx* genes is similar to that previously described for tetrapods. Conserved non-coding elements identified in bony fish were also identified in catshark *Dlx* clusters and showed regulatory activity in transgenic zebrafish. Gene expression patterns in the catshark showed that there are some expression sites with high conservation of the expressed paralog(s) and other expression sites with events of paralog sub-functionalization during jawed vertebrate diversification, resulting in a wide variety of evolutionary scenarios within this gene family.

**Conclusion:**

*Dlx* gene expression patterns in the catshark show that there has been little neo-functionalization in *Dlx* genes over gnathostome evolution. In most cases, one tandem duplication and two rounds of vertebrate genome duplication have led to at least six *Dlx* coding sequences with redundant expression patterns followed by some instances of paralog sub-functionalization. Regulatory constraints such as shared enhancers, and functional constraints including gene pleiotropy, may have contributed to the evolutionary inertia leading to high redundancy between gene expression patterns.

## Introduction

### The Osteichthyan *Dlx* Gene Family

*Dlx* genes encode a family of homeodomain transcription factors with various roles in embryogenesis, notably in many shared derived characters (synapomorphies) that evolved with the diversification of vertebrates [1]. This gene family displays a conserved genomic organization in jawed vertebrates with the clustering of *Dlx1* with *Dlx2*, *Dlx3* with *Dlx4* and *Dlx5* with *Dlx6*. The mouse is the best studied model organism in terms of the functional analysis of this family of transcription factors, with a series of mutants showing that they are often redundant. Further studies in the zebrafish have shown that most of their roles are conserved in bony vertebrates (reviewed in [2]). The earliest expression of *Dlx* genes is found in the non-neural ectoderm, including the preplacodal region, in early neurula [3]–[6]. *Dlx* genes are expressed in pre-migrating neural crest cells [5], [7] and paired sensory placodes [5], [6], [8], [9] (neural crest and sensory placodes are vertebrate synapomorphies), as well as in some migrating neural crest cells streams giving rise to neural crest cell-derived mesenchyme of the pharyngeal arches [5], [6], [10]. Associated with this expression, *Dlx* genes have been shown to have a function in the specification of neural crest cells in *Xenopus* [11], and later in the regionalization of the pharyngeal arches and their derivatives in mouse [12] and zebrafish [13] (regionalized arches are a gnathostome (jawed vertebrates) synapomorphy). *Dlx* transcription factors are expressed in the anterior brain (telencephalon and diencephalon [5], [6], [10], which are vertebrate synapomorphies) where they have a role in specifying GABA-ergic interneurons [14], [15]. In addition they are involved in the development of the sensory circuitry associated to the eyes and olfactory and otic organs [16]–[18]. Some *Dlx* genes are expressed in the later differentiating surface epiderm (arising from the gastrula non-neural ectoderm) and have been shown to play a role during papilla-derived appendage development: hair, tooth, and feather [9], [19]–[22]. Most *Dlx* genes are transcribed in the epithelial and mesenchymal compartments of the developing paired limb buds (gnathostome synapomorphies) and median fold (a vertebrate synapomorphy) [5], [6], [23]. In humans, *DLX5* and *DLX6* have been shown to be involved in a fore- and hind-limb developmental pathway activated by p63 [24] through an enhancer located more than 250 kbp away from the bigene cluster [25]. Finally, *Dlx* genes are expressed in the developing cartilage and bones (both dermal and cartilage bones [10], [26], [27], which also are vertebrate synapomorphies) where they are involved in the differentiation of chondrocytes and osteocytes [28], [29].

### Gnathostome Outgroups and Origins of the *Dlx* Genomic Organisation

**Figure 1:**
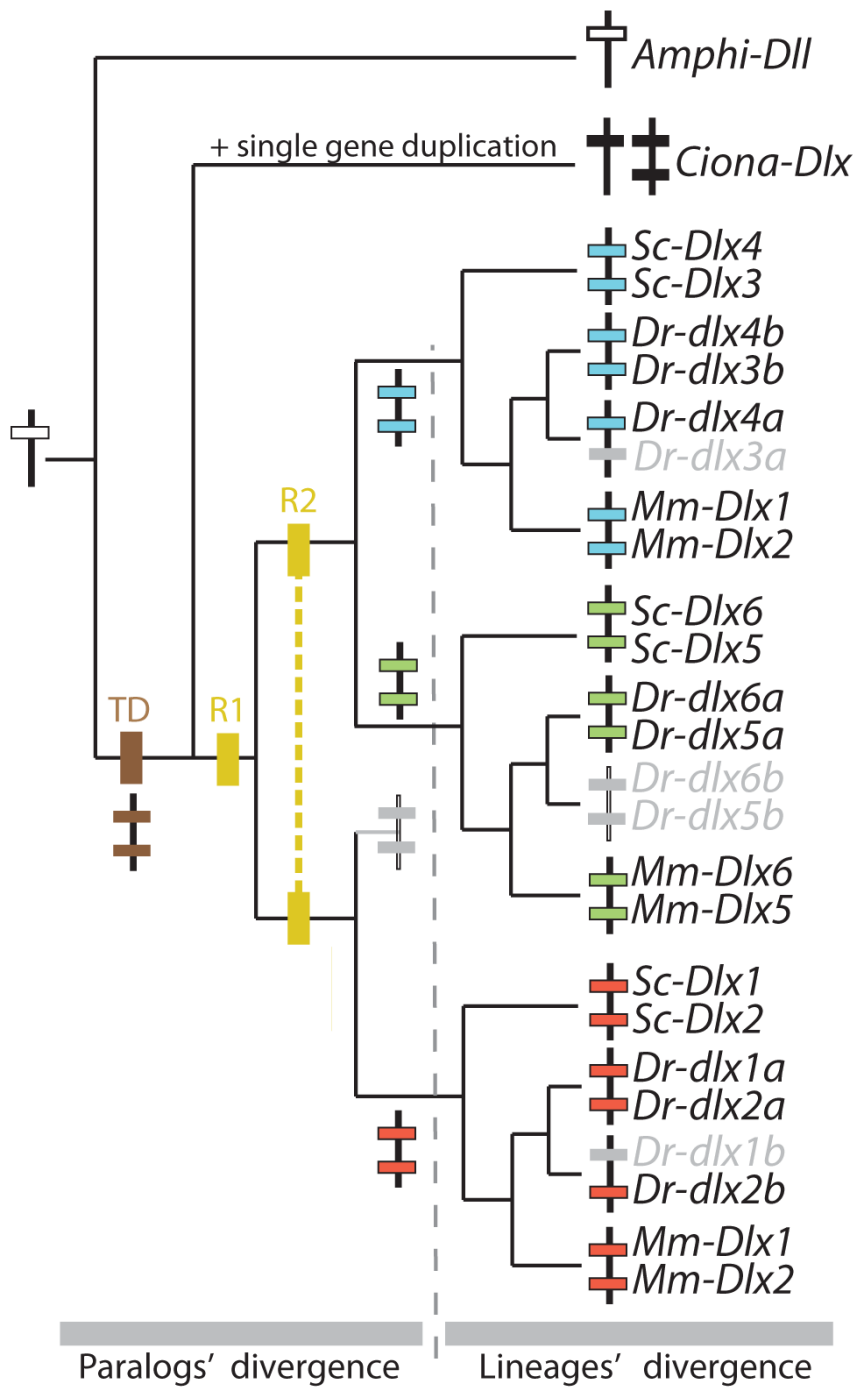
Evolutionary events leading to the extant chordate *Dlx* gene family. Phylogenetic relationships between chordate *Dlx* gene family members including: the single amphioxus Amphi-Dll gene [36]; the three *Ciona Dlx* genes: *Ciona-DllA* and *Ciona-DllB* forming a bigene cluster and single *Ciona-DllC* [35]; six *Dlx* genes from the mouse (*Mus musculus*, noted *Mm*) and catshark (*Scyliorhinus canicula*, noted *Sc*) organized as three bigene clusters: *Dlx1*–*Dlx2*, *Dlx3*–*Dlx4*, *Dlx5*–*Dlx6*. Zebrafish *dlx* genes are figured as three bigene clusters plus additional single genes (originating from the teleost-specific whole genome duplication): *dlx1a*-*dlx2a* plus *dlx2b*, *dlx3b*-*dlx4b* plus *dlx4a*, *dlx5a*-*dlx6a*. The first tandem duplication is mapped as a brown square (TD), the two vertebrate rounds of genome duplication are mapped as yellow squares annotated R1 and R2, the grey dashed line separates an early phase of *Dlx* paralog divergence before gnathostome diversification from the later phase occurring during gnathostome lineages divergence.

Within the gnathostomes, *Dlx* genes are found as three tandem bigene clusters in the genome (six coding sequences, *Dlx1* to *Dlx6*, reviewed in [30]) with additional genes (single or tandem clusters) in teleost genomes due to an ancestral whole genome duplication (*dlx1a* and *dlx1b* to *dlx6a* and *dlx6b* [31], [32]). Within the gnathostome sister group (cyclostomes), *Dlx* genes from the lamprey and hagfish have been identified but could not be strictly designated as members of the gnathostome *Dlx1* to *Dlx6* orthology groups (*Petromyzon marinus* 4 genes [1]; *Lampetra japonica*, 6 genes [33], *Eptatretus burgeri*, 6 genes [34]). Outside the vertebrates, three *Dlx* genes have been identified in the urochordate *Ciona*, two organized as a bigene tandem cluster [35], while a single gene is found in amphioxus (*AmphiDll* [36]) and most protostomes (*Drosophila melanogaster* [37], but see [38] for exceptions). The current hypothesis is therefore that there was an ancestral tandem duplication after the divergence of the cephalochordates and before the common ancestor of the urochordates and vertebrates (Figure 1), making this gene family a chordate gene family which expanded from two ancestral genes in vertebrates. This expansion is due to two series of whole genome duplications before the divergence of vertebrates [33], resulting in four bigene clusters, one being lost early, leaving three clusters in the gnathostomes: *Dlx1*/*Dlx2*, *Dlx3*/*Dlx4*, *Dlx5*/*Dlx6*. The phylogenetic relationships between those orthology groups link *Dlx1*, *Dlx4* and *Dlx6* to one of the *Dlx* gene from the ancestral single bigene cluster, while *Dlx2*, *3* and *5* are related to the other *Dlx* gene of this same ancestral cluster [30] (and see Figure 1). Because of their role in the development of key structures in vertebrate and gnathostome evolution, *Dlx* gene expression patterns have been examined extensively in lampreys and amphioxus. In the lamprey, *Dlx* genes are transcribed in the anterior brain, in the pharyngeal arches in a gnathostome-like fashion, in the olfactory and otic organs, pre-migrating and migrating neural crest cells, and the median fin fold [1], [39]. In amphioxus, *Amphi-Dll* expression was detected in a zone putatively homologous to the anterior brain within the neural vesicle, in the non-neural ectoderm during late gastrulation, and in cells associated with the photosensitive organ [36].

**Figure 2:**
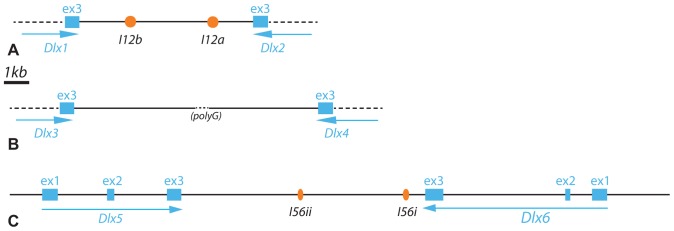
Genomic organization of the *Dlx* gene family in the catshark *Scyliorhinus canicula*. For each bigene cluster, exons are indicated as white boxes with associated number (from 1: first exon, to 3: last exon). Transcribed sequences are indicated as blue arrows, conserved non-coding elements as orange circles. Unsequenced regions are shown as dashed lines.

The lamprey *Dlx* complement has not been characterized at the genomic level, but the conservation of the bi-gene tandem clusters in bony fish has allowed comparison of the non-coding regions surrounding the *Dlx* coding sequences. Comparing mammals to zebrafish, highly conserved non-coding elements (CNEs) could be identified around the *Dlx1*/*Dlx2* pair: I12a, I12b within the intergenic sequence and URE2 upstream of *Dlx1*, and within the *Dlx5*/*Dlx6* intergenic sequence: I56i and I56ii ([39]–[41], also see Figure 2). These CNEs are considered enhancers for the *Dlx* genes as they are able to drive expression of a transgene in specific *Dlx* expressing zones of the embryo: I12a in the pharyngeal arches (mouse [41]), I12b and URE2 in the anterior brain and pharyngeal arches (mouse [42], [43] and zebrafish [44], [45]), I56i in the anterior brain and pharyngeal arches (mouse [40], [41]) or only in the anterior brain (zebrafish [46]) and I56ii in the anterior brain (mouse only [47], no enhancer activity in zebrafish [46]). A comparison of the intergenic sequence between *Dlx3* and *Dlx4* did not identify CNEs shared between the mouse and zebrafish [32] but one highly conserved element between mouse and human, I37-2, could be tested and was shown to have positive regulatory activity in the pharyngeal arches [48]. Outside the gnathostomes, no match for known CNEs could be identified in the *P. marinus* draft genome [32]. Outside the vertebrates, other regulatory sequences could be identified in *Ciona*, which were shown to be involved in driving expression in the non-neural ectoderm [49]. However the vertebrate CNEs could not be identified in *Ciona* and the *Ciona* element could not be isolated in vertebrates, suggesting that the conserved regulatory sequences obtained in bony fish were modified at some time between the “urochordates/vertebrates” divergence node and the “chondrichthyans/bony fish” divergence node.

### Vertebrate Evolution and the Chondrichthyan Lineage

Within gnathostomes, little is known about the *Dlx* genes outside the bony vertebrate group (chiefly represented by mouse and zebrafish as model organisms). The sister clade, chondrichthyans, which groups together the sharks, rays, and chimaeras, has been much less well studied in terms of embryonic development, and therefore represents an attractive branch of the gnathostome tree. Six *Dlx* genes have been identified in the leopard shark *Triakis semifasciata*, which have a usual bony fish genomic organization (at least two pairs, *Dlx1*–*2*, *Dlx5*–*6*, intergenic regions of 7–10 kb, and conserved intron-exon boundaries) [30]. We have isolated and identified 6 *Dlx* coding sequences in the small-spotted catshark *Scyliorhinus canicula* and described their expression pattern during tooth development [50], and recent thorough comparative studies of branchial arches development used *Dlx* gene expression in chondrichthyan species [51], [52]. These two studies focused on the putative role of *Dlx* gene products (and others’) in regionalizing branchial arches in the wide scheme of a Dlx code. A full description of gene expression pattern is still lacking both in terms of a wide window of organogenesis stages and in terms of global expression in the embryos. Using sequence data from the elephant shark, *Callorhinchus millii,* one *Dlx* gene pair *Dlx1*–*Dlx2* was identified on a single BAC associated with putative orthologous I12a and URE2 sequences [45]. A full view of the *Dlx* gene complement in a chondrichthyan species is therefore still lacking, in particular one which includes expression data, to enable us to propose ancestral expression domains in gnathostomes and a comprehensive view of the evolution of *Dlx* gene regulation within vertebrates. Here, we describe embryonic expression patterns over the neurulation and early organogenesis stages (from stage 16 to stage 26) and the genomic organization of the *Dlx* gene family in the catshark, and test the putative activity of some conserved non-coding elements identified around the coding sequences. These findings are analyzed in the context of gnathostome evolution, using *Ciona* and the amphioxus as outgroups. This comparative approach allows the identification of evolutionary patterns in jawed vertebrates after the series of gene duplications that occurred before jawed vertebrate evolution. In this analysis, we considered the theoretical framework of the Duplication-Degeneration-Complementation (DDC) model [53] and described occurences of (1) neo-functionalization of some paralogs by acquisition of new expression sites; (2) sub-functionalization between paralogs, through differential degeneration of the ancestral expression sites. We could characterize this sub-functionalization as total (only one member of the family has kept the ancestral expression site) or partial (more than one) but also as early (before the diversification of jawed vertebrates) or late (during their diversification). This analysis highlights very different rates of expression pattern degeneration between paralogs of one gene family, leading to heterogeneous conservation of co-expression between paralogs from one expression site to another.

## Results

### Catshark *Dlx* Genes and their Genomic Organization

Based on exons 1 and 3, six *Dlx* genes have been previously identified and assigned to *Dlx1* to *Dlx6* orthology groups [50]. We obtained four full-length cDNA sequences from cDNA librairies made from several developmental stages in the catshark [54]: *Dlx2*, *Dlx3*, *Dlx4* and *Dlx5*. Mapping the *Dlx5* cDNA sequence from the catshark and the *Dlx6* sequence from *Triakis semifasciata* [30] onto the BAC containing the *Dlx5*–*Dlx6* bigene cluster showed that the gene organization observed in all other gnathostomes was conserved in the catshark. The *Dlx5* and *Dlx6* coding regions both consist of three exons with the homeodomain coding sequence within exons 2 and 3. The STOP codons of these two genes were separated by a roughly 10 kb sequence in the BAC. Using long-range PCR we amplified the approximately 8 kb intergenic sequence between *Dlx1* and *Dlx2* (herein named SC*inter12*), and the 10 kb intergenic sequence between *Dlx3* and *Dlx4* (SC*inter34*). The intergenic regions were cloned and completely (SC*inter12*) or partially (SC*inter34*∶5 kb at the *Dlx3* end, 4.6 kb at the *Dlx4* end, poly-G in central position) sequenced using primer walking. Alignment of these intergenic sequences with mouse and zebrafish orthologs using the VISTA genome browser tool identified regions within the SC*inter12* and SC*inter56* sequences that are conserved in the gnathostomes (greater than 75% conservation over 100 bp). No similarly conserved region could be identified within SC*inter34* (see Figure S1). Conserved sequences from SC*inter12* and SC*inter56* are homologous to the putative enhancers previously characterized in mouse and zebrafish: I12a, I12b, I56i and I56ii (Table 1, Figure 2). Note that URE2 could not be identified because of its putative location outside of the *Dlx* gene cluster.

**Table 1:** Catshark CNE length and similarity defined through comparison to the mouse (*Mus musculus*) and zebrafish (*Danio rerio*) intergenic sequences.

	*Mus musculus*		*Danio rerio*		
CNE	similarity	length	similarity	Length	inserts
I12a	94%	429 bp	87%	461 bp	546 bp
I12b	72%	318 bp	72%	215 bp	480 bp
I56i	76%	338 bp	75%	285 bp	425 bp
I56ii	74%	283 bp	74%	227 bp	397 bp

### Catshark *Dlx* Gene Expression Patterns

*Dlx* probes were used to examine expression patterns of all six *Dlx* genes during the early neurulation to mid-organogenesis stages (stage 15 to 25). Hybridization using all probes designed against the six *Dlx* coding sequences resulted in a strong signal in various structures (see supplementary material in [50]) with positive expression from stage 15 to 25 for *Dlx3*, *Dlx4*, *Dlx5* and *Dlx6*; from stage 17 to 25 for *Dlx2*; and from stage 19 to 25 for *Dlx1*. The expression patterns are presented here as (i) non-neural ectoderm, sensory placodes and sensory vesicles; (ii) paired fins, median fin fold and *analia-genitalia*; (iii) anterior brain; (iv) neural crest cells and branchial arch mesenchyme. For each zone of expression, we mapped our results and those previously described from mouse, zebrafish, *Ciona* and amphioxus onto a chordate phylogenetic tree. We then proposed hypotheses to depict the evolutionary steps of *Dlx* expression patterns, both before and during the divergence of the extant gnathostomes (see Figure 1). In the case of the zebrafish data, we summed the expression patterns of both teleost-specific duplicates when necessary (*dlx4a*+*dlx4b* and *dlx2a*+*dlx2b*). As very few zones of expression are specific to only one gene of a bigene cluster, we built a tree of the clusters rather than one of the single genes in order to better show the most parsimonious evolutionary scenario.

#### (i) Non-neural ectoderm, sensory placodes and sensory vesicles

**Figure 3:**
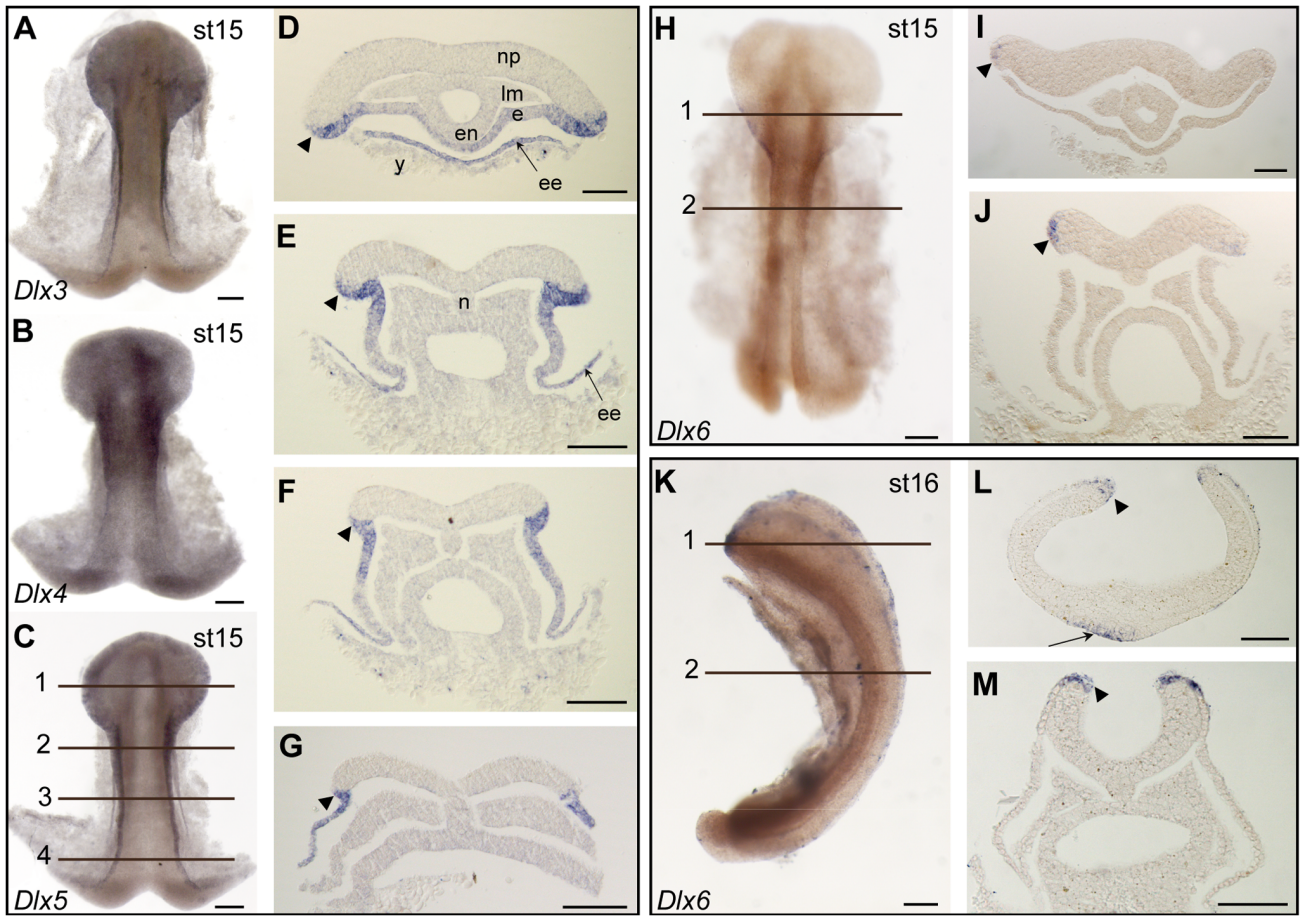
*Dlx* gene expression patterns during neurulation (stage 15–16) in the catshark. Target gene name and developmental stage are indicated for each box. Expression is evidenced by blue precipitate in cells of the non-neural ectoderm, both extra-embryonic ectoderm (ee) and embryonic ectoderm (e), including at the border of the neural plate (np, arrowhead). **A–C**: *Dlx3*, *Dlx4*, *Dlx5* expression patterns at stage 15, *in toto*; **D–G**: sections taken from embryo in C showing histological details of *Dlx5* expression pattern at stage 15. **H–J**: *Dlx6* expression pattern at stage 15. **K–M**: *Dlx6* expression pattern at stage 16. **A–C, H**: whole-mount dorsal views, anterior to the top. **D–G, I, J, L, M**: serial transverse sections, dorsal to the top; **D–G** from embryo in panel C, **D**: section 1 to **G**: section 4. **I–J** from embryo in panel H, section 1 =  panel I, section 2 =  panel J, with expression restricted to the ectoderm at the neural plate border. **K**: lateral view, dorsal to the right, anterior to the top, with location of the transverse sections, section 1 in panel L, section 2 in panel M. n: notochord; en: endoderm; lm: lateral mesoderm; y: yolk. Scale bars: A–C, H, K: 200 µm, D–G, I, J, L, M: 100 µm.

*Dlx3*, *Dlx4*, *Dlx5* and *Dlx6* transcripts were detected as early as stage 15 (early neurulation stage) in the non-neural ectoderm, on the rostral and lateral border of the neural plate (Figure 3). *Dlx6* expression was restricted to the ectodermal margin of the neural plate border (Figure 3 H–J) while *Dlx3*, *Dlx4* and *Dlx5* expression domains extended fully to the embryonic and extra-embryonic ectoderm (Figure 3 A–C and histological sections for *Dlx5* in Figure 3 D-G, histological details for *Dlx3* and *Dlx4* are not shown but were identical to *Dlx5*). During stage 16, when the neural tube starts folding, expression of *Dlx3* to *6* was still observed in the non-neural ectoderm. In particular, *Dlx6* transcripts were restricted to the folding and fusing part of the ectoderm at the neural tube border (Figure 3 K–M).

**Figure 4:**
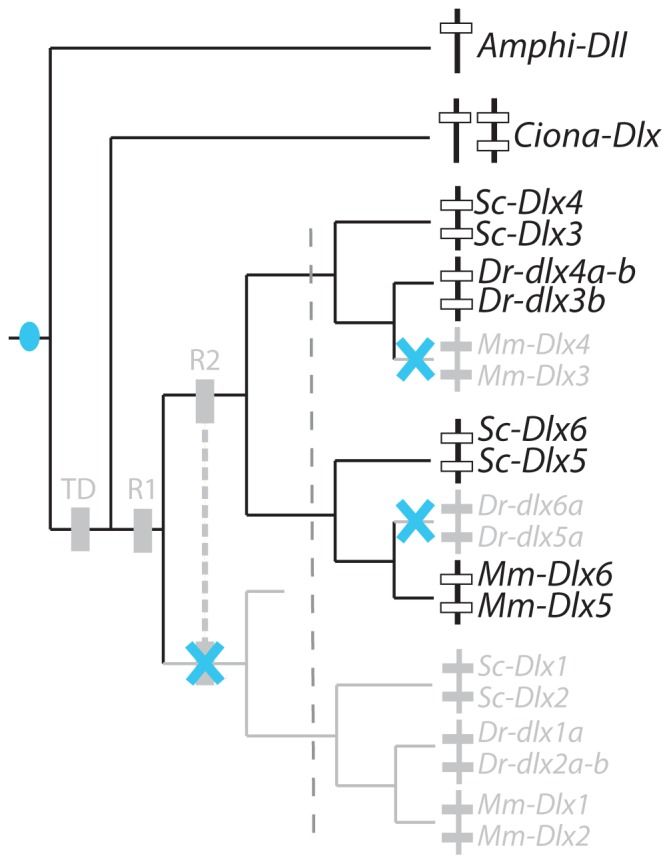
Evolutionary scenario for the expression of *Dlx* genes at the neural plate border in chordates. Genes for which expression is known to be positive are written black while genes for which no expression has been recorded are indicated in grey. The blue circle represents the hypothetical apparition of *Dlx* expression in this structure and blue cross indicates loss of expression for both *Dlx* genes of a bigene cluster. Teleost duplicates in the zebrafish were pooled together to simplify the analysis.

This first set of results of early non-neural ectodermal expression were compared to homologous expression patterns in amphioxus [36], *Ciona* [55], zebrafish [5], [6] and mouse [3], [4] and mapped onto the *Dlx* cluster tree (Figure 4). A maximum parsimony reconstruction suggests the following evolutionary scenario: ancestral *Dlx* expression in chordates was followed by several losses, one before the diversification of gnathostomes for the *Dlx1*–*Dlx2* cluster and two independent losses, one in the zebrafish lineage (loss of *dlx5a*-*6a* expression) and the other in the mouse lineage (*Dlx3*–*Dlx4*). This case therefore illustrates late (during lineage divergence) but full sub-functionalization of one ancestral expression site (neural plate border) in the mouse and zebrafish lineage (only one Dlx tandem expressed) and only partial sub-functionalization in the catshark lineage (two Dlx tandems expressed). Note that *Dlx6* expression pattern in the catshark was much more restricted but this difference was not taken into account in the analysis.

**Figure 5:**
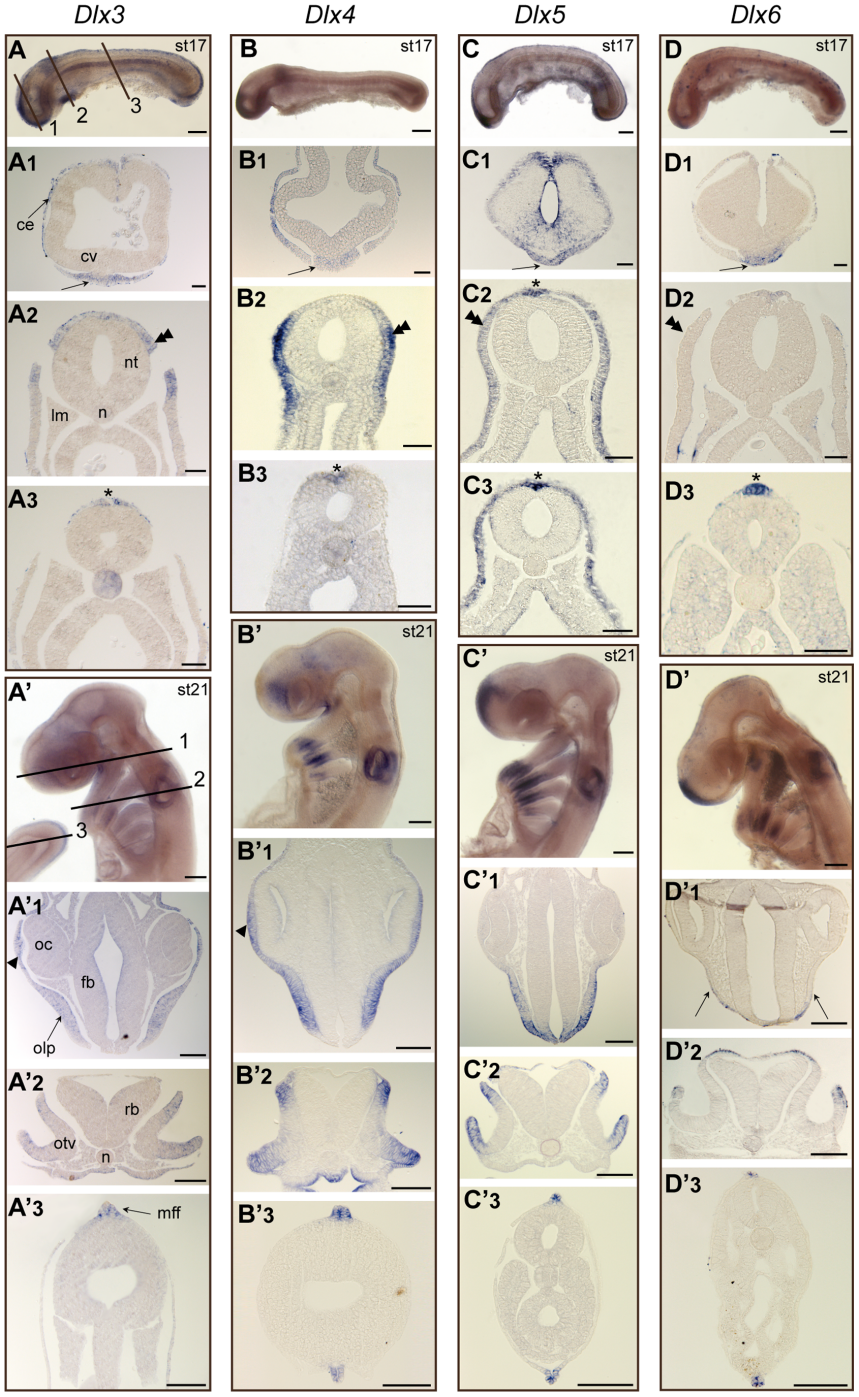
*Dlx* gene expression patterns at stage 17 and 21 in the catshark. Target gene name is indicated on top of each column, stage of development is indicated on top of each box. **A–D**, **A**′**–D**′: lateral views of whole-mount embryos; **A–D** dorsal to the top, anterior to the left; **A**′**–D**′ are anterior to the top, dorsal to the right. **A1–3, B1–3, C1–3, D1–3, A**′**1–3, B**′**1–3, C**′**1–3, D**′**1–3** are transverse sections with dorsal to the top; **A1–D1** go across the presumptive olfactory placode (black arrow) as located by section plan #1 on the A panel; **A2–D2** go across the presumptive otic placode (double arrowhead), plan #2 on the A panel; **A3–D3** go across the trunk with developing median fin fold (asterisk), plan #3 on the A panel; **A**′**1–D**′**1** go across the olfactory placode (olp) and lens placode (arrowhead), plan #1 on the A′ panel; **A**′**2–D**′**2** go across the folding otic vesicle (otv), plan #2 on the A′ panel; **A**′**3–D**′**3** go across the trunk with developing median fin fold (mff), plan #3 on the A′ panel. ce: cephalic epithelium; cv : cephalic vesicle; fb: forebrain; lm: lateral mesoderm; n: notochord; nt: neural tube; oc: optic cup; rb: rhombencephalon. Scale bars: A–D, A′–D′: 200 µm; A1–3, B1–3, C1–3, D1–3∶50 µm; A′1–3, B′1–3, C′1–3, D′1–3∶100 µm.

**Figure 6:**
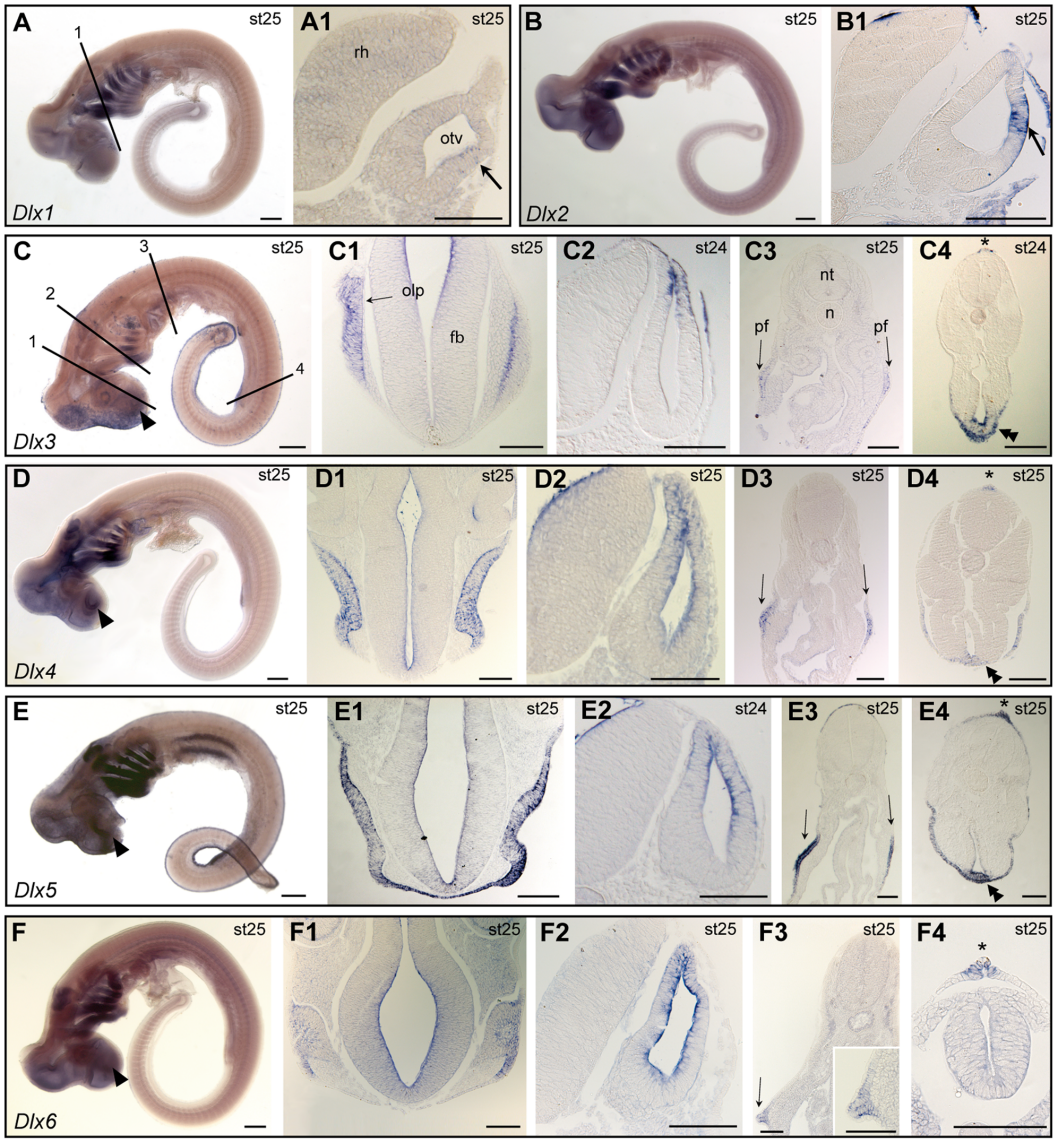
*Dlx* gene expression during early organogenesis in the catshark (stage 24–25). Target gene name is indicated in each box, developmental stage is indicated in each panel. **A–F**: lateral views of whole-mount hybridized embryos, anterior to the left, dorsal to the top. **A1, B1, C1–4, D1–4, E1–4, F1–4**: transverse sections, dorsal to the top. **A1**, **B1** are taken at the level of section plan #1 in panel A, showing the otic vesicle (otv); **C1–F1** are taken at the level of section plan #1 in panel C, showing the olfactory placode (olp); **C2–F2** are taken at the level of section plan #2 in panel C, showing the otic vesicle; **C3–F3** are taken at the level of section plan #3 in panel C, showing the presumptive pectoral fin bud (pf, arrows, higher magnification in the bottom right corner in F4); **C4–F4** are taken at the level of section plan #4 in panel C, showing the median fin fold (mff, asterisk) and the *analia genitalia* (ag, double arrowheads,except in F4). Other legends as in Figure 5. Scale bars: A–F: 400 µm; A1, B1, C1–4, D1–4, E1–4, F1–4∶100 µm.

Between stage 16 and stage 25, *Dlx3*, *4*, *5* and *6* were expressed in the cephalic ectoderm (Figure 5 and Figure 6). As early as stage 17, messenger RNAs of *Dlx3*, *4*, and *5* were detected in the developing sensory organs, in particular in the prospective olfactory and otic placodes (Figure 5 A1–2, B1–2, C1–2). At the same stage, *Dlx6* mRNAs could be detected in the presumptive olfactory region, but not yet in the presumptive otic placode (Figure 5 D1–2). Expression of *Dlx3* and *Dlx4* was also seen in the cephalic ectoderm but not in the lateral trunk ectoderm (Figure 5 A1–3 and B1–3), with expression in the lens placode at stage 21 (Figure 5 A′1 and B′1). In contrast, *Dlx5* mRNAs were detected in both the head and trunk ectoderm at stage 17 (Figure 5 C1–3). Positive staining was obtained at stage 21 for *Dlx3*, *4* and *5* probes in both thickened, well-defined, olfactory placodes and folding otic placodes (see Figure 5 A′1–2, B′1–2, C′1–2). Expression of *Dlx6* could also be detected in the most peripheral zone of the closing otic placode (Figure 5 D′2) but not in the olfactory placode although it was expressed in cephalic ectoderm anterior to the placode (Figure 5 D′1). Later expression of *Dlx3*, *Dlx4, Dlx5* and *Dlx6* could be observed at stage 25 in the folding olfactory placode and otic vesicles (Figure 6 C–F 1–2). Late expression of *Dlx1* and *Dlx2* could be detected in a lateral region of the otic vesicle starting around stage 24 (Figure 6 A1–B1).

**Figure 7:**
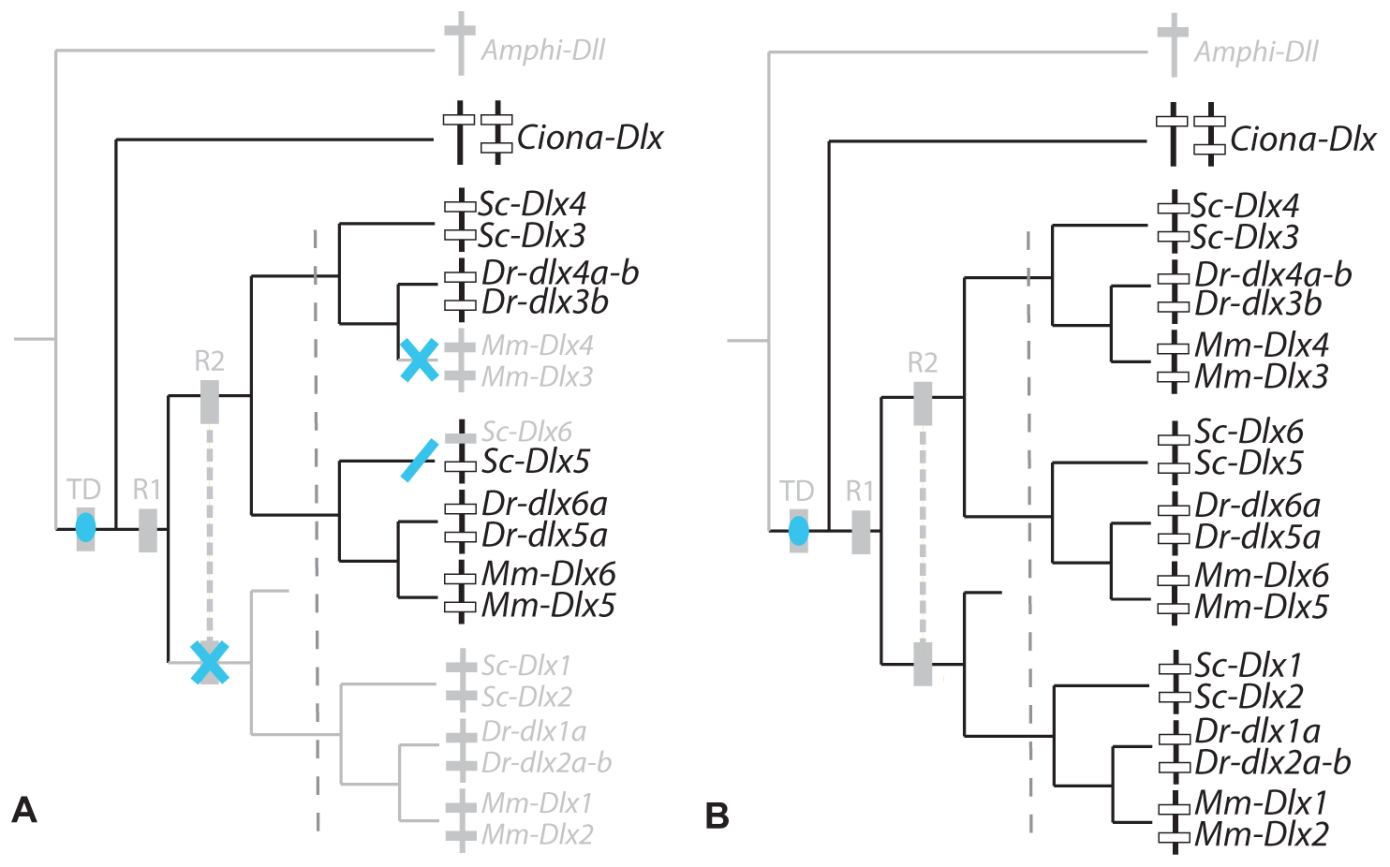
Evolutionary scenario for the expression of *Dlx* genes in developing paired sensory placodes (A) and vesicles (B). The blue circle represents the hypothetical apparition of *Dlx* expression in this structure and blue cross indicates loss of expression for both *Dlx* genes of a cluster. A tilted bar indicates when only one gene of a cluster has lost expression in his structure. Genes for which expression is known to be positive are written black while genes for which no expression has been recorded are indicated in grey.

To analyze these results within a maximum parsimony framework, we separated them into two groups: early expression in the olfactory or otic placodes (before they fold) and late expression (after folding of the placodes into vesicles). Homologous expression patterns were included from *Ciona* as it has been proposed that the paired atrial siphon primordia are homologous to vertebrate otic placodes [56]. Our data were compared with that from *Ciona*, mouse [3], [9], [57] and zebrafish [5], [6]. Sensory placodes are not found in amphioxus even though gene expression pattern data suggest that there may be homologous cell types in this organism [58]. The evolutionary scenario obtained shows ancestral expression of *Dlx* genes during the early development of paired sensory placodes, followed by early loss of expression by the *Dlx1*–*Dlx2* cluster before gnathostome diversification, and late loss of *Dlx6* expression in the catshark lineage and of *Dlx3*–*Dlx4* in the mouse lineage (Figure 7A). Full sub-functionalization is therefore observed in the mouse lineage while only partial sub-functionalization has happened in the zebrafish and catshark lineages. Late expression of all Dlx genes in paired sensory vesicles has been retained from the last gnathostome ancestor (Figure 7B) but note that expression patterns are not strictly comparable for all genes in the catshark, *Dlx5*–*Dlx6* genes are expressed in the dorsal aspects of the otic vesicle while *Dlx1*–*Dlx2* genes are localized in a more lateral aspect. The observed conserved redundancy may therefore hide more subtle sub-functionalization between paralogs in subsets of their expression patterns.

#### (ii) Paired fins, median fin fold and *analia-genitalia*

Early expression of *Dlx3*, *4*, *5* and *6* was detected in the ectodermal site of dorsal neural tube closure from the end of neurulation (stage 17, Figure 5A–D3) throughout median fin fold development (stage 21, Figure 5 A′–D′3) and at later stages (stage 25, Figure 6 C–F4). Similar expression in the ectodermal compartment of the presumptive pectoral fin bud was observed for *Dlx3*, *Dlx4*, *Dlx5* and *Dlx6* at stage 25 (Figure 6 C–F3). There was an additional site of expression of *Dlx3*, *4* and *5* at stage 25 within the *analia-genitalia* (Figure 6 C–E4).

**Figure 8:**
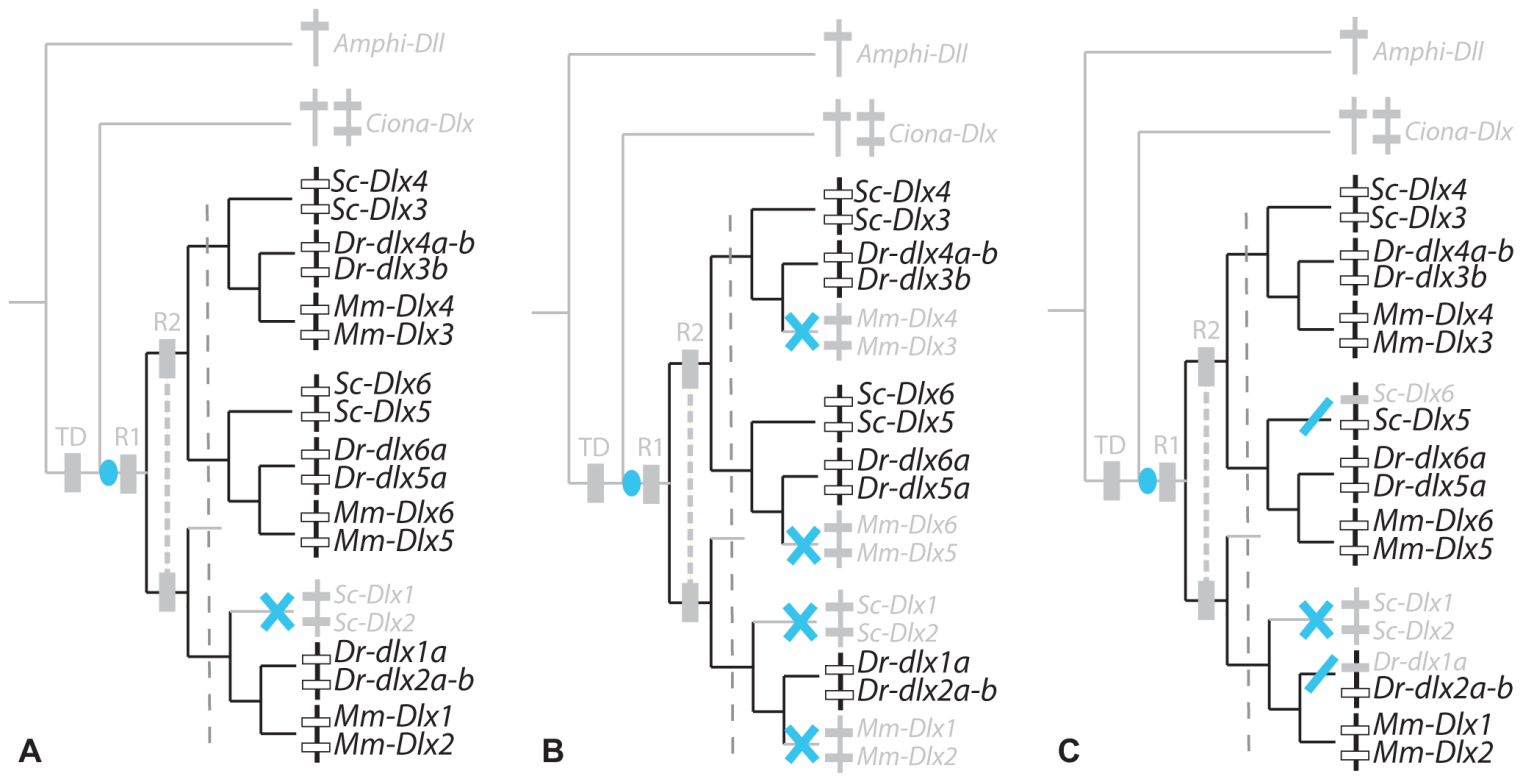
Evolutionary scenario for the expression of *Dlx* genes in fins/limbs (A), median fin fold (B) and *analia*-*genitalia* (C). See Figure 4 for legends.

Comparing these results to those from mouse and zebrafish suggests various evolutionary scenarios depending on the structure: the most parsimonious scenario (Figure 8A) for expression in the AER (in mouse, reviewed in [59]) or its homologous structure in zebrafish [6] and catshark (this study), was the recruitment of *Dlx* genes before their duplication during the two rounds of genome duplication with subsequent loss in the catshark lineage (*Dlx1*–*Dlx2* cluster). These results highlight very low (catshark) to no sub-functionalization (mouse, zebrafish) of *Dlx* genes in these structures. In the median fin fold, the scenario is identical except that all mouse *Dlx* genes have lost this zone of expression as the structure itself has disappeared from mammalian embryos (Figure 8B, expression in the zebrafish are from [6]). Finally, data for the *analia genitalia* were more difficult to compile: for the mouse, we complemented data from [60] with that from the GUDMAP Project [61] and *in situ* hybridization results at E14,5 from [62], available at MGI [63]. Together these data show that all *Dlx* genes are expressed during genital tubercle development and/or the genito-urinary system; for the zebrafish, we used data submitted by [64] in ZFIN [65] at 36hpf, showing that all *Dlx* genes except for *dlx1a* is transcribed in the developing *analia genitalia* at this stage. The scenario obtained was almost identical to that proposed for the AER zone, except for the loss of *dlx1a* expression in the zebrafish lineage and the loss of *Dlx6* expression in the catshark lineage (Figure 8C). Again, a very low level of sub-functionalization is observed for Dlx expression patterns in these structures.

#### (iii) Anterior brain

**Figure 9:**
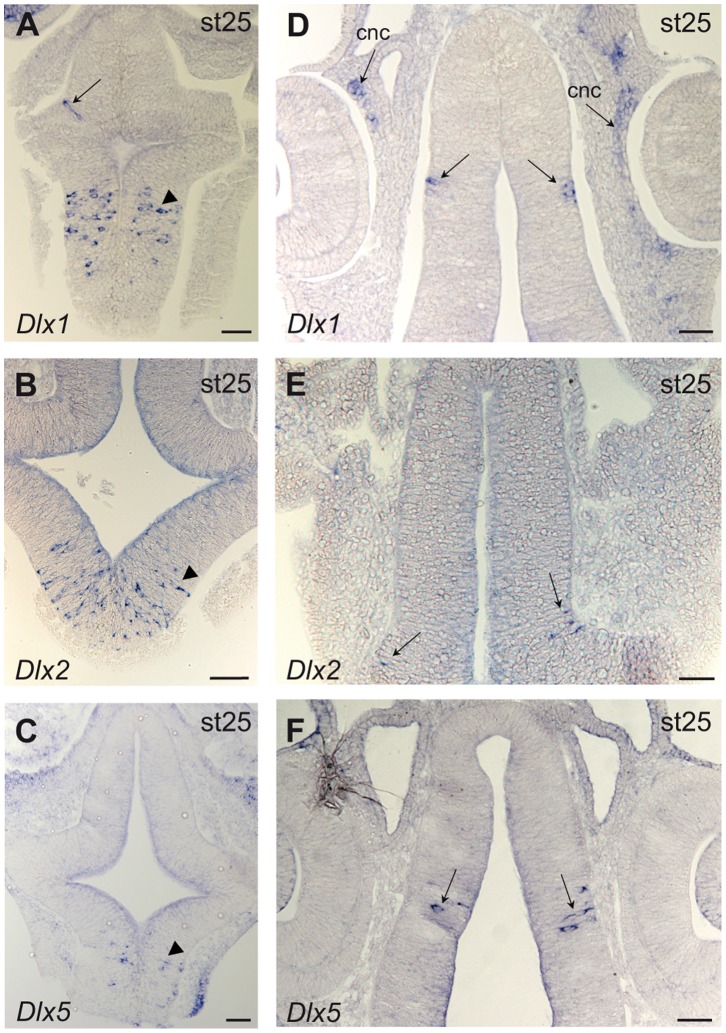
*Dlx* gene expression during brain development in the catshark. Target gene name and stage of development are indicated on each panel. Transverse sections through whole-mount hybridized embryos at stage 25, dorsal to the top, at the level of the telencephalon (**A–C**) or more posterior through the diencephalon (**D–F**). *Dlx1*, *Dlx2* and *Dlx5* are expressed in scattered cells of the telencephalon (arrowhead in **A–C**) and of the diencephalon (arrow in **A** and **D–F**). Note expression of *Dlx1* in scattered cells of the head mesenchyme at the mandibular level, interpreted as cranial neural crest cells (cnc). Scale bars: 50 µm.

*Dlx5* mRNAs were detected in the most anterior part of the neural tube at stage 17 (Figure 5 C1). During stage 21, faint expression of *Dlx5* (Figure 5C′1) and *Dlx6* (not shown) was still detected in the anterior-most part of the telencephalon at the level of the olfactory placodes and still seen until at least stage 25 (not shown). Starting around stage 25, some cells of the ventral-most part of the telencephalon (subpallium) started showing expression of *Dlx1*, *Dlx2* and *Dlx5* (Figure 9 A–C) while scattered positive cells were also observed in the diencephalon (Figure 9 A, D–F).

**Figure 10:**
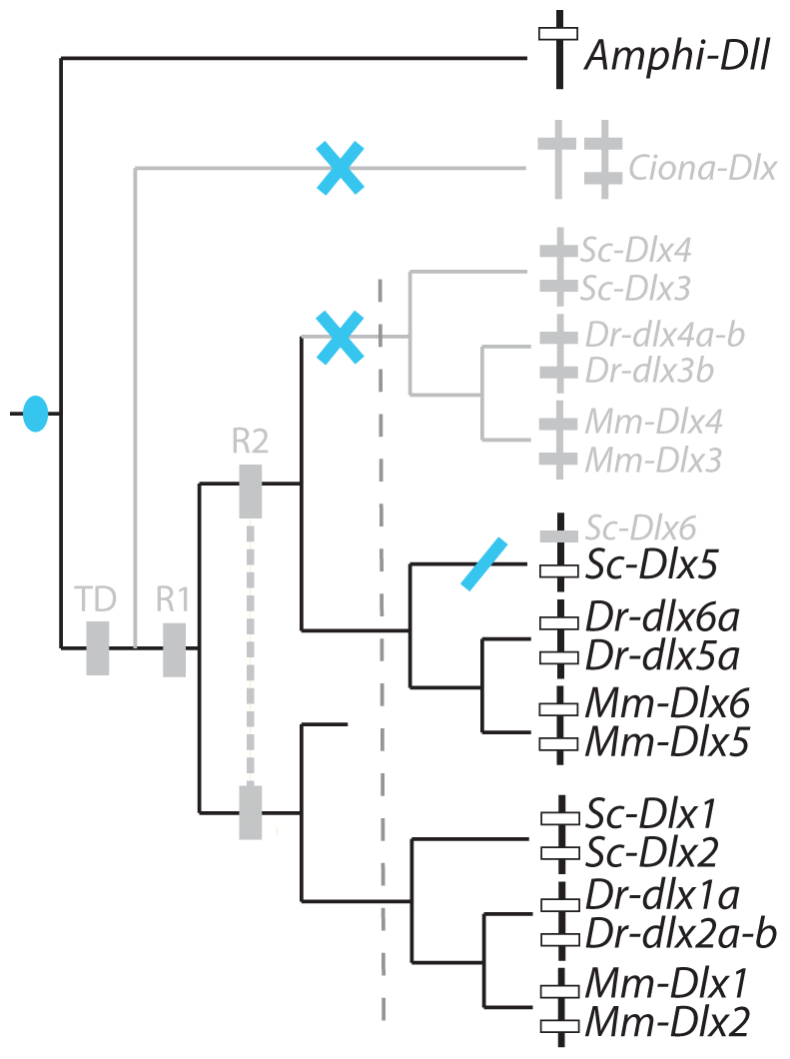
Evolutionary scenario for the expression of *Dlx* genes in anterior brain. See Figure 4 for legends.

To analyze these results, we excluded the earlier and anterior-most expression of *Dlx5* and *Dlx6* in the catshark telencephalon, because this zone of expression was not strictly homologous to those previously described in mouse and zebrafish. We only kept the later expression of *Dlx* genes in the development of the ventral telencephalon and diencephalon, and compared them to data from mouse and zebrafish (reviewed in [40] and [6]). The expression of *Amphi-Dll* has been described in an anterior subset of the cerebral vesicle [36], and was suggested to be a topological homolog to the vertebrate anterior brain [66]. We coded negative expression pattern for *Ciona* as no homologous zone of expression has been described for *Ciona Dlx* genes [35], [67]. The most parsimonious hypothesis, shown in Figure 10, is that the original expression within a chordate ancestral anterior brain territory was followed by losses for the gnathostome ancestral *Dlx3*–*Dlx4* cluster and for all *Dlx* genes in the *Ciona* lineage and finally by *Dlx6* only in the catshark. An equally parsimonious scenario would involve the convergent evolution of *Dlx* gene expression in the amphioxus cerebral vesicle and gnathostome anterior brain, followed by the same gnathostome specific losses. However, we would favor the hypothesis proposed in Figure 10 since convergent loss is more probable than convergent gain. Note that the catshark *Dlx6* loss may be artefactual as this gene is known to be expressed later than *Dlx5* in mouse and we did not screen for *Dlx6* expression later than stage 27. This scenario would involve one early (before lineage divergence) and single event of sub-functionalization with the loss of *Dlx3*–*Dlx4*. However, functional data obtained in the mouse (reviewed in [2]) and zebrafish [40], [44] show that Dlx genes are expressed during brain development with slight temporal and space differences suggesting at least partial sub-functionalization between paralogs in osteichthyans.

#### (iv) Neural crest cells and pharyngeal arch mesenchyme

**Figure 11:**
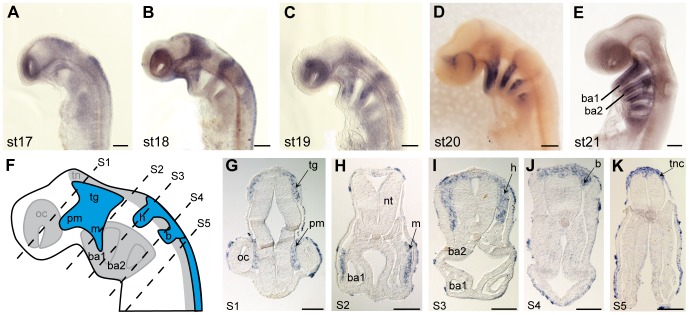
*Dlx2* expression in neural crest cells in the catshark. **A–E**: lateral views of whole-mount embryos, anterior is to the top, dorsal to the right, between stage 17 and 21. **F**: schematic of the head for embryo in panel B with location (blue colored) of the various streams of migrating neural crest cells: trigeminal (tg) stream separated in the premandibular (pm) and the mandiblar (m) streams; hyoid (h) stream; branchial (b) stream. **G–K** are transverse sections made on the embryo shown in panel B. The section level is located on panel F: G =  section 1(S1) to K =  section 5 (S5). **A**: stage 17, expression is restricted to the neural crest cells both at the cranial and trunk level. **B**: streams of cranial neural crest cells migrate ventrally: *Dlx2* expression detected in the various cranial neural crest streams and most posteriorly in the trunk neural crest cells (tnc). **D, E**: after migration, *Dlx2*-positive cells are found in the mesenchyme of branchial arches (ba). ba1: first branchial arch, ba2: second branchial arch, oc: optic vesicle, nt: neural tube. Scale bars: A–E: 200 µm; G–K: 100 µm.

*Dlx2* transcripts were detected in the cranial neural crest cells before and during (stage 17–19) their migration from the neural tube to the pharyngeal arches (Figure 11 A–E). *Dlx2* expression was localized all along the anterior-posterior axis, in a dorsal aspect of the neural tube from stage 17 to stage 19 equivalent to trunkal neural crest cells (Figure 11 A–E, K) but was then observed only in cranial neural crest cells during their migration toward the head and pharyngeal arches mesenchyme (Figure 11G–J). *Dlx2*–positive cranial neural crest cell migration was complete at stage 21. A complete description of *Dlx* gene expression in the subsequent pharyngeal arch development has been published elsewhere [52].

**Figure 12:**
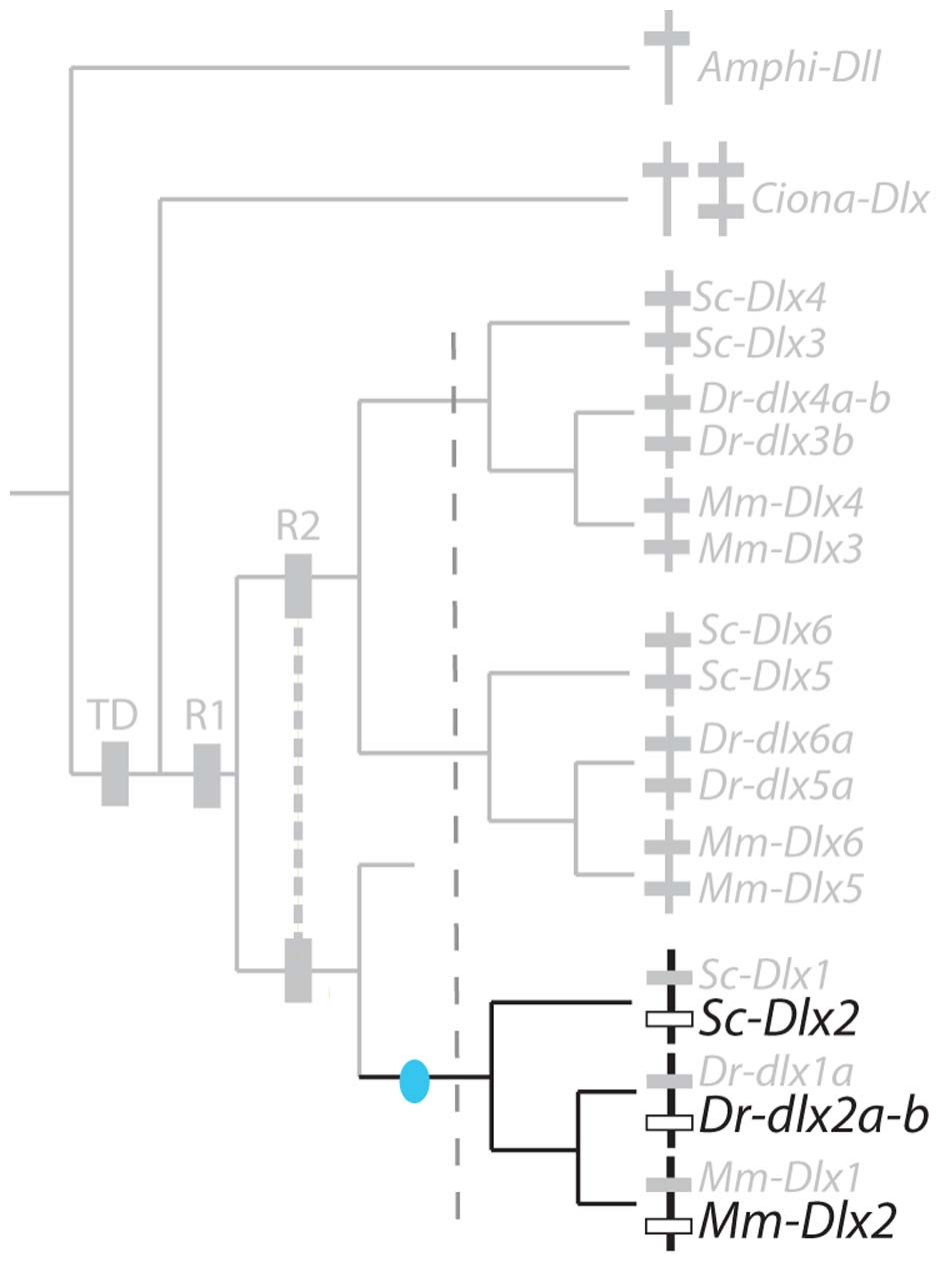
Evolutionary scenario for the expression of *Dlx* genes in premigrating and migrating neural crest cells. See Figure 4 for legends.

Of the *Dlx* genes in gnathostomes, only *Dlx2* cognates are expressed in pre-migrating and migrating neural crest cells, as described in the catshark (this work), zebrafish [5] and mouse [7]. Neural crest cells are a vertebrate synapomorphy: no homologous structure can be found in amphioxus. In *Ciona*, some cells have been proposed as the putative homologs to the vertebrate neural crest cells but they have been shown not to express *Dlx* homologs [55].The most parsimonious evolutionary hypothesis is therefore a single unique cooption of the *Dlx2* coding sequence in the development of this structure after gnathostome paralog duplication but before gnathostome divergence (Figure 12) followed by strong conservation of this expression pattern during gnathostome diversification (but see [68]). This would be the only example of neo-functionalization observed in gnathostome *Dlx* paralogs.

### Putative Catshark *Dlx* Regulatory Elements

**Figure 13:**
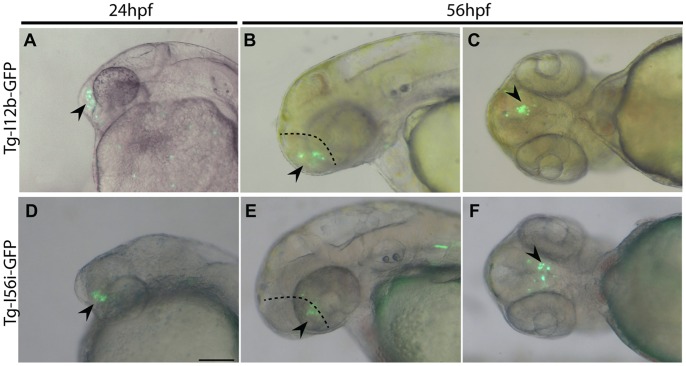
Transient transgenic expression of GFP in zebrafish embryos under catshark CNE regulation. **A–C**: GFP expression under catshark I12b regulation at 24 hpf (A, lateral view, dorsal to the top, anterior to the left) and 56 hpf (B, lateral view oriented as A; C: ventral view, anterior to the left). **D–F**: GFP expression under catshark I56i regulation at similar stage with similar orientation. The dashed line separates the telencephalon (arrowhead, with expression) from the diencephalon (no expression). Scale bar: 100 µm.

Transient transgenic zebrafish were obtained by injection of a GFP-reporter plasmid. This plasmid is negative for expression by itself but becomes active when an enhancer sequence is inserted. We built eight constructs with each of the four identified CNEs (I12a, I12b, I56i, I56ii) in both possible orientations. We obtained transient positive GFP expression in the nervous system of zebrafish embryos with the I56i and I12b sequences from the catshark genome (mean 40% positive embryos, n>200 for each CNE, n>100 for each CNE in each orientation). GFP expression could be detected with these sequences in both orientations suggesting they act as true regulatory sequences and not as promoters. Specific GFP-positive cells were observed in the anterior part of the developing brain only, in the telencephalon of embryos from 24 hours post fertilization (hpf) to 72 hpf (Figure 13 and not shown) consistent with the telencephalic expression observed at stage 25 with *Dlx1* and *Dlx2* (Figure 9). Other sites of expression observed in our description of catshark *Dlx* gene expression patterns were not sites of GFP expression in our transgenic zebrafish embryos. GFP expression was never observed with I56ii and I12a in embryos from 1 dpf until 3 dpf (n>100 for each CNE in each orientation), consistent with the absence of expression obtained with zebrafish I12a and I56ii in zebrafish transient transgenesis at early embryonic stages (not shown, n>100 embryos for each construct).

## Discussion

### Conservation of the Genomic Structure and Associated CNEs

The *Dlx* genomic organization in the catshark showed that such organization is highly conserved within the gnathostomes: that is, three bigene clusters in a tail-to-tail arrangement, including an approximately 10 kb intergenic sequence with two highly conserved non-coding regions between *Dlx1* and *Dlx2* (I12a and I12b) and between *Dlx5* and *Dlx6* (I56i and I56ii). This organization is in clear contrast with what is currently known of the lamprey genome: using gnathostome CNE sequences, we failed to retrieve any similar sequence from the lamprey genome (Ensembl, Pmarinus_7.0) and *Dlx* coding sequences were very difficult to assign to any of the gnathostome orthology groups in both the lamprey [33] and hagfish [34]. The age of the first gnathostome fossils are estimated at 435 Ma while the first vertebrate forms are thought to occur at 470 Ma [69]. Whatever the actual time point when the vertebrate/gnathostome *Dlx* bigene clusters have become stable in terms of genome structure, these data suggest a very high level of functional constraint on both the structural arrangement of the *Dlx* genes and on the intergenic CNE sequences at least over the past 430 Ma. Previously, similar results in other vertebrates were considered good examples of highly conserved gene regulatory blocks linking developmental genes to their shared enhancers [40], [70].

Our transient transgenesis assays demonstrated that enhancer activity in the brain is conserved for catshark I12b and I56i CNEs when tested in zebrafish (Figure 13), as has been previously shown for homologous sequences from zebrafish and mice [40], [41], [43], [44], [46]. However, major technical advances are still necessary to test the enhancer activity of these sequences within catshark embryos to support these results and describe their activity *in situ*. The results we obtained in zebrafish suggest that enhancer activity is limited to the brain (telencephalon), which is a region of endogenous *Dlx1*, *Dlx2* and *Dlx5* expression. This observation was consistent with what has been previously described in mice and zebrafish as well as what has been obtained with the elephant shark *Dlx* CNEs [45]. However, the transgene expression pattern covers only a very small subset of *Dlx* gene expression patterns at the observed stages, suggesting other regulatory sequence may be in charge of transcription at these sites. Further identification of additional regulatory sequences associated with the *Dlx* bigene clusters is needed to shed light on the molecular events involved in the evolution of *Dlx* expression patterns.

### Single Gene *vs* Cluster Regulation

Enhancer sharing by two genes of the same cluster has been proposed to explain both the conservation of the genomic organization and overlapping *Dlx* gene expression [40]. However, the detailed and complete expression patterns we describe here show clear differences between the *Dlx* clusters (Table 2). Expression of *Dlx3* and *Dlx4* were identical (over the observed stages of development and at the level of precision of an *in situ* hybridization) while there were clear differences between *Dlx5* and *Dlx6* expression patterns: *Dlx6* was repeatedly found expressed within a subset of the *Dlx5* spatial expression pattern, notably in the folding zones of organs such as the neural plate border, olfactory and otic placodes (see Figure 3, 5, 6). *Dlx1* and *Dlx2* expression patterns were more restricted and showed both zones of co-expression (late expression in the otic vesicle, telencephalon) and in one case, *Dlx2* specific expression in the cranial and trunkal neural crest cells before or during their migration. The hypothesis of shared enhancers for both genes of a bigene cluster in the catshark therefore seems applicable for most *Dlx* genes. The *Dlx5*–*Dlx6* situation suggests an additional hypothesis: as *Dlx6* was expressed within a subset of the *Dlx5* expression pattern, and notably often in the folding/fusing zones, there may be a shared enhancer for this cluster with an additional restricting regulatory sequence specifically linked to *Dlx6*. This putative regulatory situation in the catshark is concordant with proposed evolutionary scenarios in which there is a lineage-specific loss of *Dlx6* expression in the early otic placode, paired and median fins, anterior brain and restriction of expression in the non-neural ectoderm, late otic and olfactory placode development. This scenario also supports the hypothesis that there originally was co-expression of *Dlx5* and *Dlx6* driven by a shared enhancer, followed by lineage-specific addition of a *Dlx6* negative regulator modulating the original expression pattern.

**Table 2:** Summary of *Dlx* expression patterns between stage (st) 15 and st25 in the catshark.

	*Dlx1*	*Dlx2*	*Dlx3*	*Dlx4*	*Dlx5*	*Dlx6*
nne *st15*			+	+	+	+ fold
Pres. olp *st17*			+	+	+	+
olp *st18–25*			+	+	+	+ fold
otp *st18*			+	+	+	
otv *st19–25*	+*	+*	+	+	+	+ fold
Ventral tel. *st25*	+	+			+	
Diencephalon *st25*	+	+			+	
mff *st17–25*			+	+	+	+
pf *st25*			+	+	+	+
*Analia genitalia st25*			+	+	+	
NCC *st17–18*		+				
Branchial arches	+	+	+	+	+	+

Positive expression is shown with a +. Stage of expression indicated in first column. +fold indicates when expression is found restricted to the folding part of an organ. mff: median fin fold; NCC: neural crest cells; nne: non-neural ectoderm; olp: olfactory placode; olv: olfactory vesicle; otp: otic placode; otv: otic vesicle; pf: pectoral fin; Pres.: presumptive. Asterisk is for specific *lateral* expression of *Dlx1* and *Dlx2*.

### Heterogeneous Rates of Sub-functionalization among Expression Sites

The catshark gene expression patterns described here show both similarities and differences with those of other gnathostomes. Reconstruction of ancestral states with maximum parsimony resulted in various evolutionary scenarios depending on the expression site involved. Only one case of neo-functionalization is highlighted with our results: *Dlx2* expression in premigratory and migratory neural crest cells. This novelty originated before gnathostomes diversified and was highly conserved in all three lineages analyzed (Figure 12). Other expression sites were all inherited from the ancestral Dlx bigene cluster. We describe one scenario with no detectable sub-functionalization (late expression of all *Dlx* genes in the paired sensory placodes, Figure 7B) and two scenarios with weak sub-functionalization (loss of *Dlx1*–*Dlx2* expression only in paired fins/limbs (Figure 8A) and the same scenario with additional loss of catshark *Dlx6* and zebrafish *dlx1a* expression in *analia genitalia* (Figure 8C)). On the other hand, other sites of expression show more extensive paralog sub-functionalization: expression in the non-neural ectoderm (Figure 4), early paired sensory placode development (Figure 7A), expression of all but *Dlx3*–*Dlx4* in the developing forebrain (Figure 10).

Lineage-specific evolutionary events may explain other peculiar losses: for example, the case of the *Dlx3*–*Dlx4* cluster is well-known in the mouse (and most probably represent the situation in all mammalian species) where there has been both recruitment of these genes in placenta development [71], [72], and a specific case of protein function modification for *Dlx4*, from a nuclear transcription factor to cytoplasmic protein [73]. Modification of *Dlx* expression patterns is therefore probably not independent in early developing paired sensory placodes and neural plate border, while placental expression may be considered as a modified “non-neural ectoderm expression” at the neurula stage. Another case of non-independency in our evolutionary scenarios is the loss of mouse *Dlx* expression during median fin fold development, because of the simple loss of this structure, even at embryonic stages, in mammals (Figure 8B). Finally we observe a very similar set of evolutionary scenarios in the case of limb/median fin fold/*analia genitalia*, with redundant expression patterns for all *Dlx* genes except in the case of the catshark *Dlx6* and *Dlx1*–*Dlx2* cluster (Figure 8). This observation is consistent with the hypothesis of a common gene regulatory network which may have been coopted in early vertebrates from the median fin fold towards the lateral mesoderm to make the paired appendages [74]. A common gene regulatory network has also been proposed for the development of both distal limb and genitalia [75] which would explain overall similarities in the evolution of *Dlx* expression patterns in these three structures.

In all cases, the evolutionary scenarios proposed here involve much fewer evolutionary steps than the one we previously proposed for *Dlx* expression in gnathostome teeth [50] where the discrete expression pattern in tooth buds are very different when the two genes of a cluster are compared, and also very different from one lineage to another. This difference may be because of the detail in which we looked at expression patterns (for example, here we coded dorsal expression of *Dlx3*–*6* and the lateral expression of *Dlx1*–*2* in the otic vesicle in a similar way). However, the low conservation of *Dlx* expression pattern in teeth and scales is most probably the result of complete independency of gene expression pattern between genes of a single cluster, therefore also showing extensive independent changes in expression patterns during vertebrate evolution [50]. These variations in the pace of change in gene expression patterns may therefore be dependent on: (i) the putative functional redundancy within the family (as in Figures 4 and 7A) as opposed to strict specificity of the paralog(s) involved in one zone (as in Figures 10 and 12), and (ii) the possibility of each gene of a cluster evolving independently or not, which may be more likely for some structures than for others (previously discussed).

### Evolution of Regulatory Sequences *vs* Evolution of Expression Patterns

As major players in chordate development and in vertebrate and gnathostome morphological novelties, *Dlx* genes have been extensively examined in model species and gnathostome outgroups. With the description of the *Dlx* gene complement in the catshark, we obtained several evolutionary scenarios for *Dlx* gene expression modification before and after the vertebrate whole-genome duplications. These results show that there are some morphological regions with highly conserved gene expression within the gnathostomes, such as migrating neural crest cells, late sensory placodes and brain either because of early sub-functionalization (before lineage divergence) or because of non-sub-functionalization. The two former zones of expression have not been linked yet to any putative regulatory sequence while the latter has been linked to gnathostome conserved non-coding elements which have highly conserved regulatory activity in mouse, zebrafish and possibly in the catshark. In contrast, expression in the gnathostome neural plate border showed that there was sub-functionalization after the second round of whole-genome duplications and during gnathostome diversification. One enhancer element was identified just upstream of the *Ciona DllB* promoter and shown to drive expression in the non-neural ectoderm. Both results are congruent with the presence of a non-neural ectoderm enhancer linked to the ancestral *Dlx* cluster which may have then been lost differentially in various lineages, and whose sequence has evolved significantly during urochordate and gnathostome evolution. Finally, two sets of results suggest ancestral involvement of *Dlx* genes in gnathostome early placode and fin/limb/*analia genitalia* development, with secondary losses in the different lineages. No enhancer has been described for the regulation of early placode expression, but the regulation of *Dlx5* and *Dlx6* in the mammalian limb has been proposed to act through a promoter as well as a long-range enhancer (located more than 250 kb upstream of *Dlx6*) in a response to p63 binding [24], [25]. Sequence conservation for this long-range enhancer appears to be restricted to amniotes (see Supplementary Figure 8 in [25]), though, as suggested by our evolutionary scenario, the enhancer activity may be conserved within gnathostomes.

### Conclusion

The Duplication-Degeneration-Complementation model [53] suggests that there is sub-functionalization of each paralog after duplication and deep evolutionary time. This has been reported for several gnathostome homeobox gene families with expression pattern complementation between duplicates or loss of one of the duplicates [76]. However, our description of gnathostome *Dlx* expression patterns depicts various evolutionary scenarios including frequent conservation of the ancestral expression pattern for all, or most, duplicates (e.g. in fins, sensory placodes/vesicles) leading to high redundancy in paralog expression patterns. These observations may be interpreted as a sign of functional complementation between paralogs though functional studies of *Dlx* genes tend to show redundancy between them instead [8], [12]. This situation with multiple redundant paralogs has been observed in other homeobox families within the gnathostomes, such as the *Hox* genes [77]. *Hox* genes are also organized as clusters with shared regulatory sequences, and also are highly pleiotropic genes. We interpret these data as a consequence of an “evolutionary inertia” where the genomic structure, presence of shared regulatory sequences, and gene pleiotropy, act as a brake which strongly slows down the evolutionary process of degeneration and complementation among paralogs.

## Materials and Methods

### Ethic Statements

For the zebrafish experiments, we obtained a permit for protocol #BL-256 from the Ottawa University Animal Care Committee. The manipulations on zebrafish were preformed according to guidelines from the Canadian Council for Animal Care. Manipulations on catshark embryos were all performed after euthanasia with MS222 so that potential suffering was minimized. No permit was needed for catshark manipulations because they were limited to early embryonic stages, all less than stage 26.

### cDNAs and BAC Sequences

*Dlx* gene sequences from *Triakis semifasciata* were used to design degenerate primers to amplify partial catshark cDNA sequences (for details see [50]) and were compared using BLAST to the 5 cDNA libraries obtained from catshark embryos (for details see [54]) to obtain full-length sequences. Putative exons 1 and 3 of each six *Dlx* coding sequences have previously been amplified from genomic DNA and then assigned to each of the six gnathostome *Dlx* orthology groups [50]. The amplified exons 1 and 3 were then used to probe a BAC library of the catshark genome (see [54] for details) with radiolabelled probes. One clone was isolated and tested positive for the presence of *Dlx5* and *Dlx6*. Another clone was tested positive for the presence of *Dlx4* only. The BAC clone containing *Dlx5* and *Dlx6* was sequenced by standard shotgun sequencing method at the Genoscope (France).

### Long-range PCR

Intergenic regions linking *Dlx1* to *Dlx2* (SC*inter1–2*) and *Dlx3* to *Dlx4* (SC*inter3–4*) were amplified using the Expand Long Template PCR System with System 3 (Roche). Primers were designed in the end of the coding sequence of the third exon (SC*inter1–*2: Dlx1.1 GAAACAGGGTAATGGTGCATTAGAGAACAGCGC, Dlx2.1 CCCGCGGCCTTTATAGGGAACTACTCCTG; SC*inter3–4*: Dlx3.1 CGATGGAGCACAGTCCCAACAACAGCG, Dlx4.1 CACCTGCCAACCTCCGCTGCTTTGTC). The following cycling conditions were used; for SC*inter1–2*, initial denaturation 92°C, 2′, followed by 10 cycles of denaturation (D) 92°C, 20″, annealing (A) 68°C, 30″, elongation (E) 68°C, 15′, followed by an additional 25 cycles of: D- 92°C, 20″, A- 68°C, 30″, E- 68°C, 15′ 20″ and final E- 68°C, 7′; for SC*inter3–4:* initial D- 92°C, 2′, followed by 10 cycles D- 92°C, 20″, A- 64°C, 30″, E 68°C, 15′, followed by an additional 20 cycles D- 92°C, 20″, A- 64°C, 30″, E- 68°C, 15′ 20″ and a final E- 68°C, 7′. Starting volumes of PCR reactions were 35uL for SC*inter1–2* and 200uL for SC*inter3–4*. PCR reactions were purified using the High Pure PCR Template Preparation Kit (Roche), precipitated using standard methods and resuspended in 25 µL 1xTE. Purified PCR reactions were polished and 5′-phosphorylated using the Expand Cloning Kit (Roche), purified again using the High Pure PCR Template Preparation Kit (Roche), precipitated using standard methods and resuspended in 10 µL 1xTE. 125 ng (SC*inter1–2*) or 300 ng (SC*inter3–4*) of purified, polished and 5′phosphorylated DNA was inserted into Vector I, packaged into the λbacteriophages and used to infect *E.coli* DH5α using the Expand Cloning Kit (Roche). Positive clones were selected, purified using the Wizard Plus Minipreps DNA Purification System (Promega) and screened for clones with the correct insert size by restriction digest with *Not*I using standard methods.

A primer walking approach was then used to sequence both intergenic sequences, leading to a full sequence of about 10 kb between *Dlx1* and *Dlx2*, and a partial intergenic sequence of about 9.6 kb between *Dlx3* and *Dlx4* (two stretches of poly-G blocked further sequencing in the centre of the intergenic sequence, 5 kb could be sequenced at the *Dlx3* end, 4.6 kb at the *Dlx4* end, poly-G in central position).

### VISTA Alignments

The sequenced intergenic sequences were aligned against their orthologs in mouse (*MMinter12*, *MMinter34* and *MMinter56*) and zebrafish (*DRinter1a2a*, intergenic region between *dlx1a* and *dlx2a*; *DRinter3b4b*, intergenic region between *dlx3b* and *dlx4b*; *DRinter5a6a*, intergenic region between *dlx5a* and *dlx6a*) retrieved from the Ensembl genome browser data (respectively NCBIM37 and Zv9). The alignment was done using the mVISTA tool from the Vista Genome Browser [78], with a threshold of 70% similarity over 100 bp.

### Catshark Embryo Staging

Catshark (*Scyliorhinus canicula*) embryos were obtained from the Station de biologie marine (Roscoff, France, CNRS and MNHN). All embryos were maintained at 17°C in sea water until dissection and staging after [79]. They were dissected and then fixed for 48 hours at 4°C in a phosphate buffered saline solution containing 4% paraformaldehyde. Embryos were then dehydrated in methanol and stored at −20°C.

### Catshark *Dlx* Probes and *in situ* Hybridization

*Dlx* probes were designed as described in [50]. Antisense RNA digoxigenin-UTP probes were transcribed using SP6 or T7 RNA polymerases (Roche), according to the orientation of the insert in the plasmid. *In situ* hybridisations on catshark embryos were performed according to standard methods with modifications described in [80]. Proteinase K treatments (10 µg/ml) were adapted for different embryonic stages: 15 min at room temperature for young embryos (before stage 20), 30 min for older embryos. The colour detection step was performed using the NBT-BCIP reaction. Embryos were post-fixed in 4% PFA after whole mount *in situ* hybridisation, then cleared and stored in glycerol at 4°C until photographed. Whole-mount hybridized embryos were put through several baths of absolute ethanol, then in butanol and finally embedded in paraplast for 10 µm cross-sections. Negative whole-mount detections were also verified after histological sections.

### Transient Transgenesis in Zebrafish

Zebrafish were raised at 28° C under a 14∶10 hour light-dark cycle as previously described [81]. Transgene constructs were produced following [45]: each conserved non-coding element (CNE) was inserted into the multiple cloning site of a vector that contained a β-globin minimal promoter-GFP cassette, in both directions. This insert is located immediately upstream of the β-globin-GFP fragment and the resulting CNE-β-globin-GFP DNA fragment is flanked at both ends by Tol2 recombinase recognition sequences. The primers used to amplify the CNEs from the intergenic sequences (either a DNA preparation of the *Dlx5*–*Dlx6* BAC clone, or of the subcloned *Dlx1*–*Dlx2* intergenic sequence obtain by long-range PCR) were designed against sequence 30 to 50 bp upstream of the conserved sequence: scI56i-F = GCCATGGGTCTGATCTCATT; scI56i-R = TCAGCTTGGCACTTTCACTG (425 bp amplified); scI56ii-F = TAACCGGACCGAAGAGTGAG; scI56ii-R = CCTTTTGCCTCCCATTTCTT (397 bp amplified); scI12a-F = AAACGGCTCAAAATCAGGAG; scI12a-R = TCCGGAATCCTGGATAGTCA (546 bp amplified); scI12b-F = TTCTGCCAAAAGCTCCAAAT; scI12b-R = TTGCAATGGTTGACATCTCTG (480 bp amplified) (see Figure S1 for the location of each primer). Around 70 ng/µl of the transgene construct was injected into fertilized zebrafish embryos at the one-cell stage and resulting fluorescence was observed after 24 hours under a fluorescent microscope (Nikon NBZ 1500 dissecting microscope). Results were comparable whatever the orientation of the CNE sequence in the construct.

## Supporting Information

Figure S1 mVISTA alignments of the catshark Dlx intergenic sequences with their mouse and zebrafish orthologs. Each of the catshark intergenic sequences, SC*inter*1–2 (top), SC*inter*3–4 (middle) and SC*inter*5–6 (bottom), are aligned against the mouse (alignments 1) and the zebrafish (alignments 2) orthologous regions. The transcribed sequence of each gene is indicated (arrow). The alignment was done using the mVISTA tool from the Vista Genome Browser, conservation level is shown as a curve along the alignment. Beyond the chosen threshold (70% similarity over 100 bp), conserved regions within the coding sequences are shown in blue, transcribed non-coding regions in white, un-transcribed non-coding regions in red. Primers used to amplify the conserved putative regulatory regions are indicated on top of the alignments (arrowheads).
(TIF)
