# Supplementary figures and images for: Heterogeneous Conservation of Dlx Paralog Co-Expression in Jawed Vertebrates
Source: PLoS One. 2013 Jun 28;8(6):e68182. doi: 10.1371/journal.pone.0068182 (PMC3695995; doi:10.1371/journal.pone.0068182)

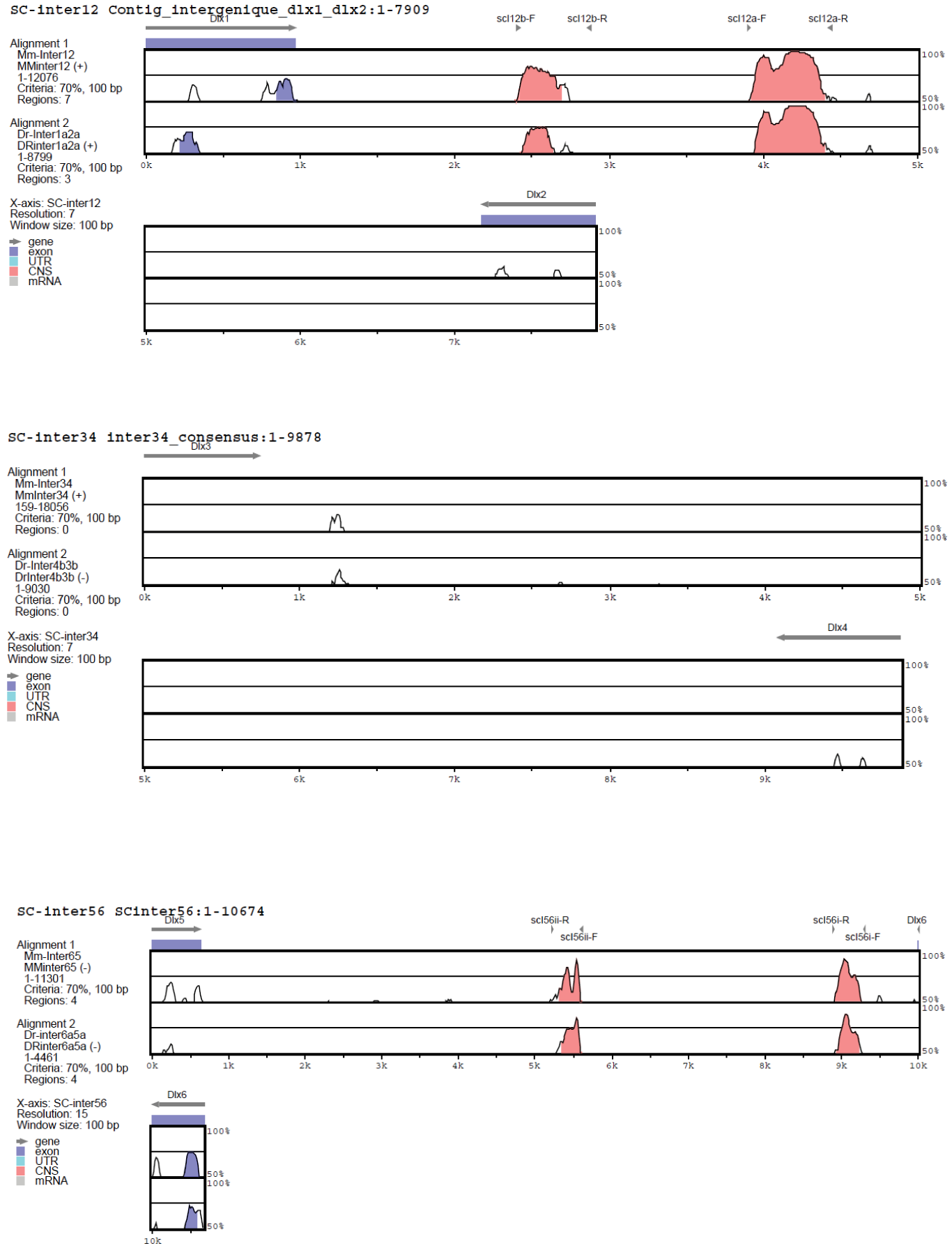

Supplement: Figure S1 — mVISTA alignments of the catshark Dlx intergenic sequences with their mouse and zebrafish orthologs. Each of the catshark intergenic sequences, SCinter1–2 (top), SCinter3–4 (middle) and SCinter5–6 (bottom), are aligned against the mouse (alignments 1) and the zebrafish (alignments 2) orthologous regions. The transcribed sequence of each gene is indicated (arrow). The alignment was done using the mVISTA tool from the Vista Genome Browser, conservation level is shown as a curve along the alignment. Beyond the chosen threshold (70% similarity over 100 bp), conserved regions within the coding sequences are shown in blue, transcribed non-coding regions in white, un-transcribed non-coding regions in red. Primers used to amplify the conserved putative regulatory regions are indicated on top of the alignments (arrowheads). (TIF) [file pone.0068182.s001.tif]
